# Generation and application of avatars in pharmacometric modelling

**DOI:** 10.1007/s10928-023-09873-9

**Published:** 2023-07-24

**Authors:** Estelle Chasseloup, Andrew C. Hooker, Mats O. Karlsson

**Affiliations:** Department of Pharmacy, Uppsala University, Box 580, Uppsala, 75123 Sweden

**Keywords:** Pharmacometrics, Avatars, Digital twins, Virtual twins, Clinical endpoint

## Abstract

Simulations from population models have critical applications in drug discovery and development. Avatars or digital twins, defined as individual simulations matching clinical criteria of interest compared to observations from a real subject within a predefined margin of accuracy, may be a better option for simulations performed to inform future drug development stages in cases where an adequate model is not achievable. The aim of this work was to (1) investigate methods for generating avatars with pharmacometric models, and (2) explore the properties of the generated avatars to assess the impact of the different selection settings on the number of avatars per subject, their closeness to the individual observations, and the properties of the selected samples subset from the theoretical model parameters probability density function. Avatars were generated using different combinations of nature and number of clinical criteria, accuracy of agreement, and/or number of simulations for two examples models previously published (hemato-toxicity and integrated glucose-insulin model). The avatar distribution could be used to assess the appropriateness of the models assumed parameter distribution. Similarly it could be used to assess the models ability to properly describe the trajectories of the observations. Avatars can give nuanced information regarding the ability of a model to simulate data similar to the observations both at the population and at the individual level. Further potential applications for avatars may be as a diagnostic tool, an alternative to simulations with insurance to replicate key clinical features, and as an individual measure of model fit.

## Introduction

The concept of digital twins was initially developed for the aerospace industry as a way to mirror a real life system (e.g., a plane or any part of it such as an engine) [1, 2]. The model integrates historical and real time data to enable informed and tailored predictions under multiple conditions. The idea was generalized to the in-silico representation of an industrial process, hence applicable in almost all the industrial areas [3, 4]. The concept is now also well established in health, engineering, and systems biology where it refers to the creation of a digital representation of a physical or biological system in order to explore and understand its behavior. The terms virtual twin or avatar are commonly used as well.

For biological systems, any simulation from a model of the system can be considered a digital twin, if it shares feature(s) with a real-life counterpart, and are broadly used in clinical pharmacology. As application examples one can cite: (1) dose adjustments using physiologically based pharmacokinetic (PBPK) models [5, 6]; (2) simulation of virtual trials with different protocols using a comprehensive physiology based mathematical model and patient-level clinical data [7]; (3) investigation of the interface between physiology-based/pharmacokinetics/pharmacodynamics models and personalized clinic-genomic data [8]; (4) predicting the occurrence of adverse events using quantitative systems toxicology (QST) models [9]; (5) creating a virtual twin under placebo conditions for each treated volunteer [10] or a virtual placebo cohort [11] using generalized linear models with conditional means; or (6) identifying subgroups with enhanced treatment effect with regression trees [12]. Most of these examples requires bottom-up approaches, generating system-information-driven models (i.e., PBPK, QST models), or lack a proper inter-individual variability (IIV) description. Thus, while there are many examples of the use of avatars in clinical pharmacology, there is, to our knowledge, not any investigation of its use with pharmacometric models for longitudinal data developed in a data-driven fashion and focusing on IIV. Such population models and their simulations have critical applications in drug discovery and development [13–16].

In this work we define avatars as individual simulations matching clinical criteria of interest compared to observations from a real subject within a predefined margin of accuracy. Such samples from the model parameters probability density function (PDF) could have interesting properties as they are selected for the closeness of their associated time-course profile to individual observations. Potential application are model diagnostics, alternative to classical model simulations when a model cannot be improved, insurance to filter out unreasonable simulated individual profiles, and individual information about agreement with the model.

Two previously published models were used as illustrative examples: a model for hematological toxicity (HT) [17], and a multivariate model for glucose homeostasis (IGI) [18]. For these two models avatars were selected among model simulations according to the closeness of the simulated individuals to some clinically relevant criteria using different combinations of avatar selection criteria (nature and number of clinical criteria, accuracy of agreement, and number of simulations). Some tools to assess the performances of the selection process are proposed to evaluate the impact of each avatar setting.

## Material and methods

NONMEM [19] version 7.4.4 was used to simulate profiles from the two example models. R [20] version 3.6.0 and bash were used to post-process the results and hence perform the avatar selection. Throughout the paper model simulations refers to individual predictions (IPRED), without residual variability. The simulations were performed with the design and the covariates of the realized data set, like simulations used for creation of visual predictive checks [21].

### Selection of avatars

**Fig. 1:**
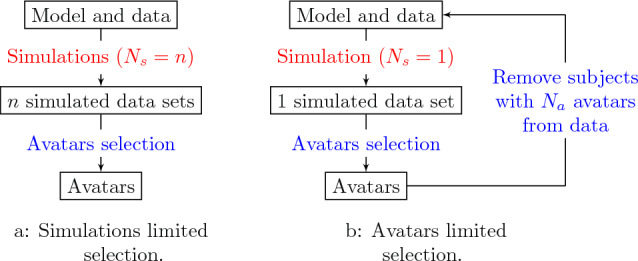
Illustration of the avatar selection workflow depending on the selection method used. Red steps were performed by Nonmem, blue steps were performed using bash and R (Color figure online)

For a given observed individual *i*, an avatar is a simulated profile, i.e., a predicted time profile from a sampled vector of individual random effects, for which all clinical criteria of interest fall within a specified accuracy. Accordingly, to decide on whether a simulation is an avatar for a given observed individual one needs to define a set of clinical criteria of interest, abbreviated *CC* (e.g., $C_{\max}$, $C_{\min}$, baseline value), and their associated accuracy of agreement, abbreviated *AA* (e.g., $\pm 15\%$ or $\pm 5$ concentration units). For each clinical criteria of interest *j*, the accuracy of agreement interval is computed for each individual *i* as $CC_{AA,ji}=CC_{ji,obs}\pm AA$, where $CC_{ij,obs}$ is the observed clinical criteria of interest *j* for the individual *i*. In addition to these two avatar settings (clinical criteria of interest and accuracy of agreement), the number of simulations is also a critical parameter as it will impact the final number of simulations matching the avatars settings. Therefore, one can define two methods to generate avatars (see Fig. 1): (a) a simulation limited selection method where the total number of simulations (e.g., $N_s=1000$) is fixed, a priori before the selection process, or (b) an avatars limited selection method where it is the number of avatars per subject that is defined a priori (e.g., $N_a=1$). In the latter case, the simulations are performed one by one and the individuals are removed from the simulation data set as their simulations match the avatars settings. Consequently, an avatar simulation efficiency ratio may be computed for each subject to quantify the number of simulations required for a given subject to match the avatars settings. The avatar simulation efficiency ratio is computed as follows: $r=N_a/N_s$, where $N_s$ is the number of simulation that was necessary for the subject *i* to match the $N_a$ requirement. In this work $r=1/N_s$ since $N_a$ was always set to 1.

These “avatar settings" (i.e., clinical criteria of interest, accuracy of agreement, and selection method) are defined more specifically for the illustrative examples in the section below about models and designs.

### Models and designs

Two application examples are used: a hemato-toxicity (HT) model and an integrated glucose-insulin (IGI) model.

The HT model describes one dependent variable, the neutrophil count, after an intravenous administration of docetaxel in twenty-four phases II studies fora total of 601 patients. Patients received a dose of 75 or 100 mg/m$^2$, most of them as a 1-h infusion, but for a few patients, two or three short infusions were given [17, 22]. The subjects not complying with the avatar selection criteria described in the next section were removed from the analysis, i.e., subjects with less than three samples or missing a proper baseline assessment (25 individuals).

The IGI model describes six dependent variables involved in the glucose homeostasis: glucose (Glu), insulin (Ins), glucagon (Gln), C-peptide, Glucagon-like Peptide 1, and Glucose-dependent Insulinotropic Polypeptide [18]. In this work, we focused on Glu, Ins, and Gln, and only used their related observations [23]. The data were obtained after oral glucose tolerance tests. Sixteen subjects(8 healthy controls and 8 type II diabetes patients) were included in the study and given three glucose doses (25 g, 75 g, and 125 g).

### Specific criteria for avatar selection for the two examples

The clinical endpoints used to select avatars from this system were neutrophil counts: (i) before the first administration or baseline(B), (ii) at maximal depletion or nadir (N), and (iii) at the expected time of the next administration or return (R), which typically is set at three weeks. The clinical criteria chosen to select avatars for the IGI model, for which the data were log-transformed, were the natural logarithm of the maximum observed concentration ($C_{\max}$) for each of the three measurement types: $Glu_{\max}$, $Ins_{\max}$, and $Gln_{\max}$. Avatars were searched for each dose independently for the IGI model, it resulted in a data set of 48 subjects.

For the HT model two different relative symmetric AA were tried ($\pm 5\%$, and $\pm 25\%$). In the IGI model the log-transformed data were modeled, hence a relative symmetric AA ($\pm 25\%$) and an absolute asymmetric AA ($\pm 0.29$ log-transformed observation units) were tried. The latter corresponding to an asymmetric 1.33-fold AA interval on the untransformed data.

For the HT and the IGI models the avatar selection process was made for each clinical criterion independently and for different criteria combinations, with different accuracy of agreement, and with different selection methods. The simulations limited selection method was used for both models while the avatars limited selection method was applied only to the IGI model, to keep the number of avatars settings investigated to a reasonable amount. For the avatars limited selection method, the required number of avatars per individual was set to 1 in all the selection settings presented in Table 1.

**Table 1:** Avatars settings for the two example models

Clinical criteria	Selection limitation	Accuracy of agreement
Hemato-toxicity model (HT)		
N	1000 simulations	±5%
B, N	1000 simulations	±5%
B, N, R	1000 simulations	±5%
N	1000 simulations	±25%
B, N	1000 simulations	±25%
B, N, R	1000 simulations	±25%
N	10,000 simulations	±5%
B, N	10,000 simulations	±5%
B, N, R	10,000 simulations	±5%
N	10,000 simulations	±25%
B, N	10,000 simulations	±25%
B, N, R	10,000 simulations	±25%
Integrated glucose-insuline model (IGI)		
Glu	1 avatar	±0.29
Glu, Ins	1 avatar	±0.29
Glu, Ins, Gln	1 avatar	±0.29
Glu	1000 simulations	±25%
Glu, Ins	1000 simulations	±25%
Glu, Ins, Gln	1000 simulations	±25%
Glu	1000 simulations	±0.29
Glu, Ins	1000 simulations	±0.29
Glu, Ins, Gln	1000 simulations	±0.29
Glu	10,000 simulations	±25%
Glu, Ins	10,000 simulations	±25%
Glu, Ins, Gln	10,000 simulations	±25%
Glu	10,000 simulations	±0.29
Glu, Ins	10,000 simulations	±0.29
Glu, Ins, Gln	10,000 simulations	±0.29

*B* baseline, *N* nadir, *R* return, *Glu* glucose, *Ins* insulin, *Gln* glucagon

### Avatars analysis

To explore the properties of the samples from the model distribution retained by the avatar selection process some numerical and graphical analysis were performed. The time profiles were examined individually to assess whether the avatars were closer to the observations than the whole set of simulations.

For each avatar settings the distribution of the number of avatar per subject was graphically explored using violin plots and numerically by computing its average, standard deviation, minimum and maximum value. The number of subjects without avatars was also reported (total number and percentage).

The distributions of the individual samples obtained by the avatar selection were examined to determine whether the restrictions imposed at the individual level to retain only the closest simulations to the observed profiles induced observable differences. To do so, the three distributions of (1) the avatars samples, (2) the posterior EBEs, and (3) the theoretical distribution from the model were superposed. One avatar per subject was selected randomly for the subjects with multiple avatars to avoid an over representation of these subjects in the avatar distribution. The distributions of the observed endpoints, simulated endpoints and avatars endpoints were also compared to each other to evidence any observable discrepancies.

The relative difficulty of each clinical criterion was explored, together with the increase in difficulty between a clinical criterion alone and the combination of three clinical criteria. The metric used to assess the difficulty was the individual smallest absolute difference between the observation and the prediction across 1000 simulations, for the criteria of interest when assessing only one criteria, or for the worst criteria when assessing a combination.

Finally, in order to quantify if the avatars were closer to the initially observed profiles than all the simulations, the root mean squared error (RMSE) was computed separately for all the simulations and the subset of avatars, for all the time points and at the clinical criteria of interest, according to equation 1:

1 $$\begin{aligned} {RMSE = \sqrt{\frac{1}{n_{tot}}\sum _{i=1}^{N} \sum _{j=1}^{n_i}(Obs_{ij} - Pred_{ij})^2}} \end{aligned}$$

where $Obs_{ij}$ is the observation of individual *i* at time *j* and $Pred_{ij}$ the corresponding individual prediction, *N* is the number of individuals, $n_i$ is the number of observations per individual and $n_{tot}$ is the total number of observations.

## Results

Avatars were generated for the HT and the IGI model using the avatar settings described in Table 1. Tables 2 and 3 give statistics about the number of avatars per subject obtained for each avatar setting. All these statistics decreased when the avatars settings are stricter: increasing number of clinical criteria, lower accuracy of agreement, or lower number of simulation. The inverse tendency was observed for the number of individual without avatars, this metric increased when the avatars settings were stricter.

**Table 2:** Results for each setting used to generate avatars for the two example models using the simulation limited method

Criteria	Number of simulations	Agreement accuracy	Avatars per subject		
			Mean (SD)	Min–Max	Subjects with no avatar
Hemato-toxicity model (HT)					
N	1000	±5%	24.7 (16.2)	0–95	21 (3.6%)
B, N	1000	±5%	2.3 (5.9)	0–79	192 (33.1%)
B, N, R	1000	±5%	0.2 (0.6)	0–6	523 (90.2%)
N	1000	±25%	123.2 (77.6)	0–427	6 (1%)
B, N	1000	±25%	49.1 (45.7)	0–427	32 (5.5%)
B, N, R	1000	±25%	20.5 (25.7)	0–202	113 (19.5%)
N	10,000	±5%	245.6 (155.4)	0–942	3 (0.5%)
B, N	10,000	±5%	23.7 (57.9)	0–785	51 (8.8%)
B, N, R	10,000	±5%	1.8 (4.9)	0–51	387 (66.7%)
N	10,000	±25%	1233.9 (770.1)	0–4477	2 (0.3%)
B, N	10,000	±25%	494 (455.7)	0–3900	12 (2.1%)
B, N, R	10,000	±25%	206.2 (258.6)	0–2269	69 (11.9%)
Integrated glucose-insuline model (IGI)					
Glu	1000	±25%	424 (171.5)	0–697	1 (2.1%)
Glu, Ins	1000	±25%	377.7 (164.2)	0–694	1 (2.1%)
Glu, Ins, Gln	1000	±25%	268.9 (227.6)	0–677	4 (8.3%)
Glu	1000	±0.29	566.9 (256)	46–934	0 (0%)
Glu, Ins	1000	±0.29	123.9 (95.3)	0–345	1 (2.1%)
Glu, Ins, Gln	1000	±0.29	39.1 (40.2)	0–131	7 (14.6%)
Glu	10,000	±25%	4258.7 (1709.2)	0–6891	1 (2.1%)
Glu, Ins	10,000	±25%	3795.4 (1634.2)	0–6827	1 (2.1%)
Glu, Ins, Gln	10,000	±25%	2674.8 (2297.4)	0–6805	4 (8.3%)
Glu	10,000	±0.29	5676.4 (2564.9)	444–9333	0 (0%)
Glu, Ins	10,000	±0.29	1248.9 (952.3)	8–3294	0 (0%)
Glu, Ins, Gln	10,000	±0.29	391.1 (402.7)	0–1369	5 (10.4%)

*B* baseline, *N* nadir, *R* return, *Glu* glucose, *Ins* insulin, *Gln* glucagon, *SD* standard deviation

**Table 3:** Results for each setting used to generate avatars using the avatar limited method

Criteria	Number of avatar	Agreement accuracy	Simulation efficiency ratio		Subjects with no avatar
			Mean (SD)	Min–Max	
Integrated glucose-insuline model (IGI)					
Glu	1	±0.29	0.667 (0.370)	1–54	0 (0%)
Glu, Ins	1	±0.29	0.351 (0.366)	1–1516	0 (0%)
Glu, Ins, Gln	1	±0.29	0.0705 (0.151)	1–7766	3 (6.25%)^a^

^a^Simulations were stopped after 23,579 iterations

*Glu* glucose, *Ins* insulin, *Gln* glucagon, *SD* standard deviation

**Fig. 2:**
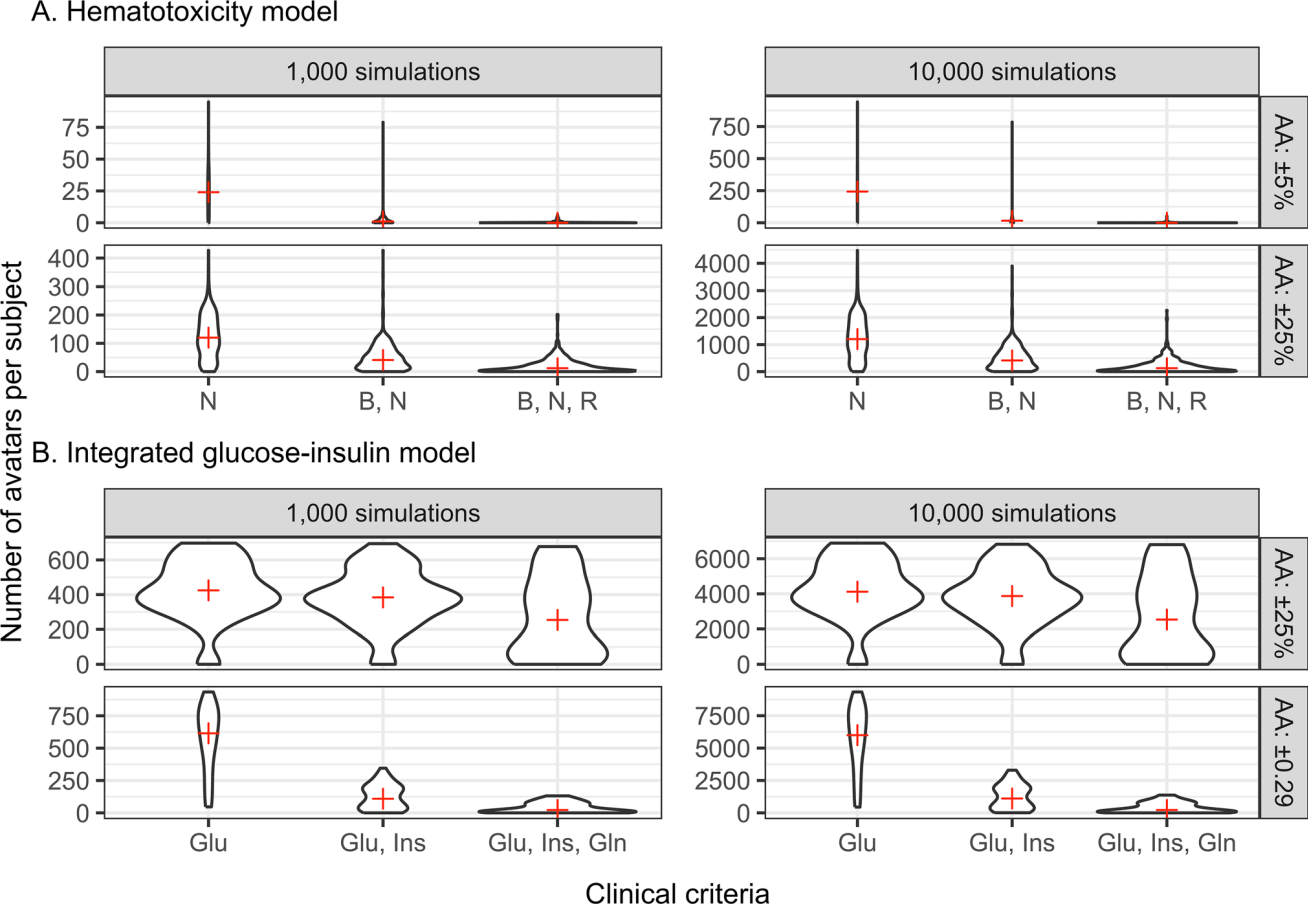
Violin plots showing the distribution of the number of avatars per subjects for the two example models. Red crosses indicate the median of the distribution. *AA* acuracy of agreement, *N* nadir, *B* baseline, *R* return, *Glu* glucose, *Ins* insulin, *Gln* glucagon (Color figure online)

Violin plots (Fig. 2) show the distribution of the number of avatars per individual for all the scenarios limited by simulations investigated. The distribution were flattened by stricter accuracy of agreement, or left truncated with increasing number of clinical criteria. Larger number of simulations scaled the distribution proportionally over a wider range. A graphical illustration of the avatars selection for two random individuals for the two example models is available in the Appendix 6. The figures show that the avatars’ time-profiles are closer to the individual observed time profile at least at the time corresponding to the clinical endpoint of interest used during the selection. Increasing number of clinical criteria to match increased the closeness of the avatars to the observed profiles over the different dependent variables, and the number of simulations required to get an avatar (decreasing avatar simulation efficiency ratio *r*).

**Fig. 3:**
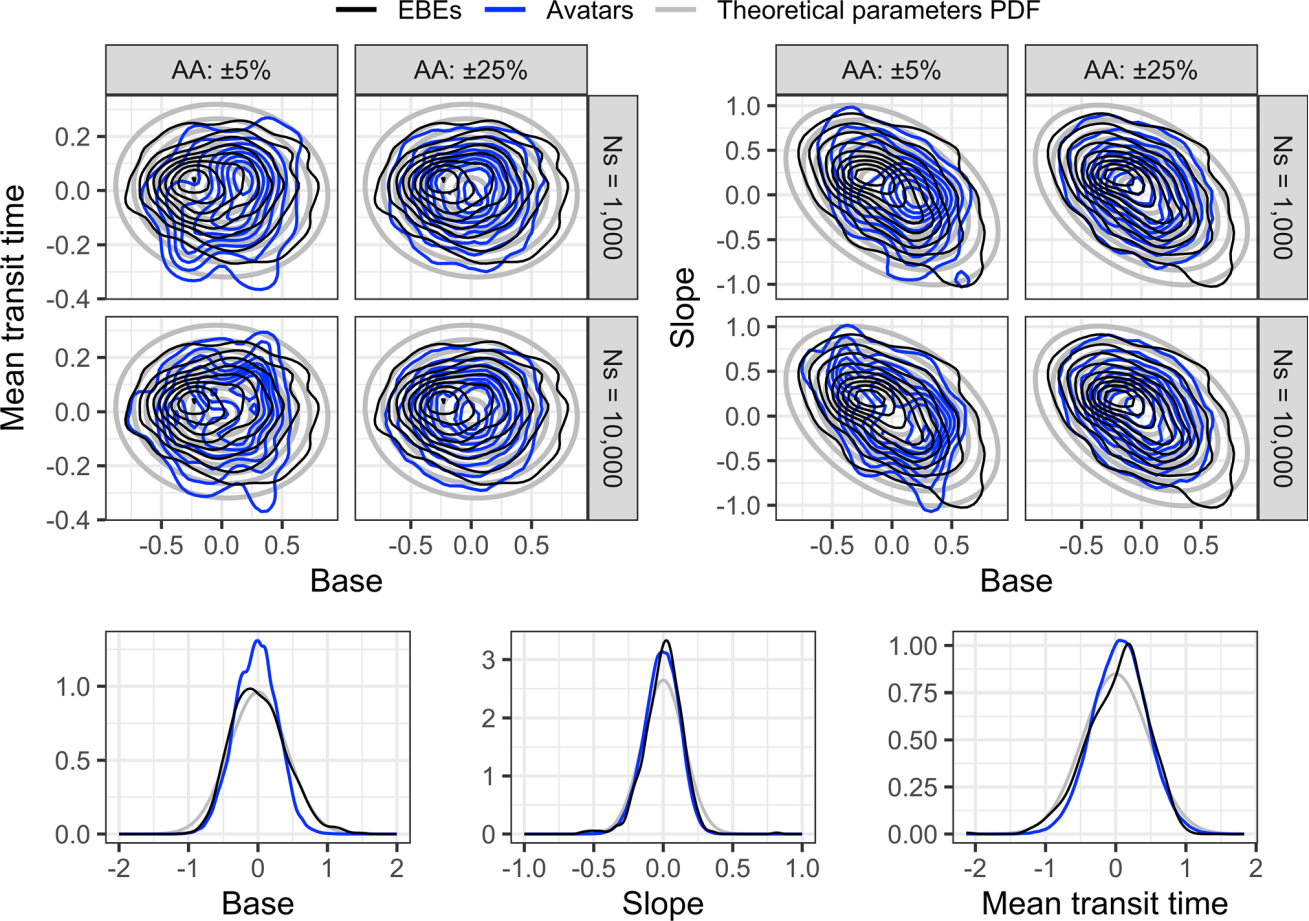
Overlay of the theoretical inter-individual variability distributions, posterior EBEs, and EBEs samples chosen by the avatars selection process for the parameters of the HT model, using three clinical criteria. The upper right panel presents the base and the slope joint distribution, while the upper left panel presents the base and the mean transit time joint distribution. The bottom panel represents the three marginal distributions. One avatar was selected randomly per subject. *AA* accuracy of agreement, *EBEs* empirical Bayes estimates, *PDF* probability density function, *Ns* number of simulations

The bivariate distribution plots in Fig. 3 represent the IIV of the parameters two-by-two for the HT model in the upper part, and the marginal distributions in the bottom part. The plots are an overlay of the model theoretical PDF of the parameter?s IIV distribution, the posterior EBEs, and the avatar samples. The three distribution overlapped reasonably well, both the EBEs and the avatars samples distributions were slightly shrunken, and the center of the EBEs distribution deviated slightly from the center of the theoretical distribution, contrary to the avatars samples.

**Fig. 4:**
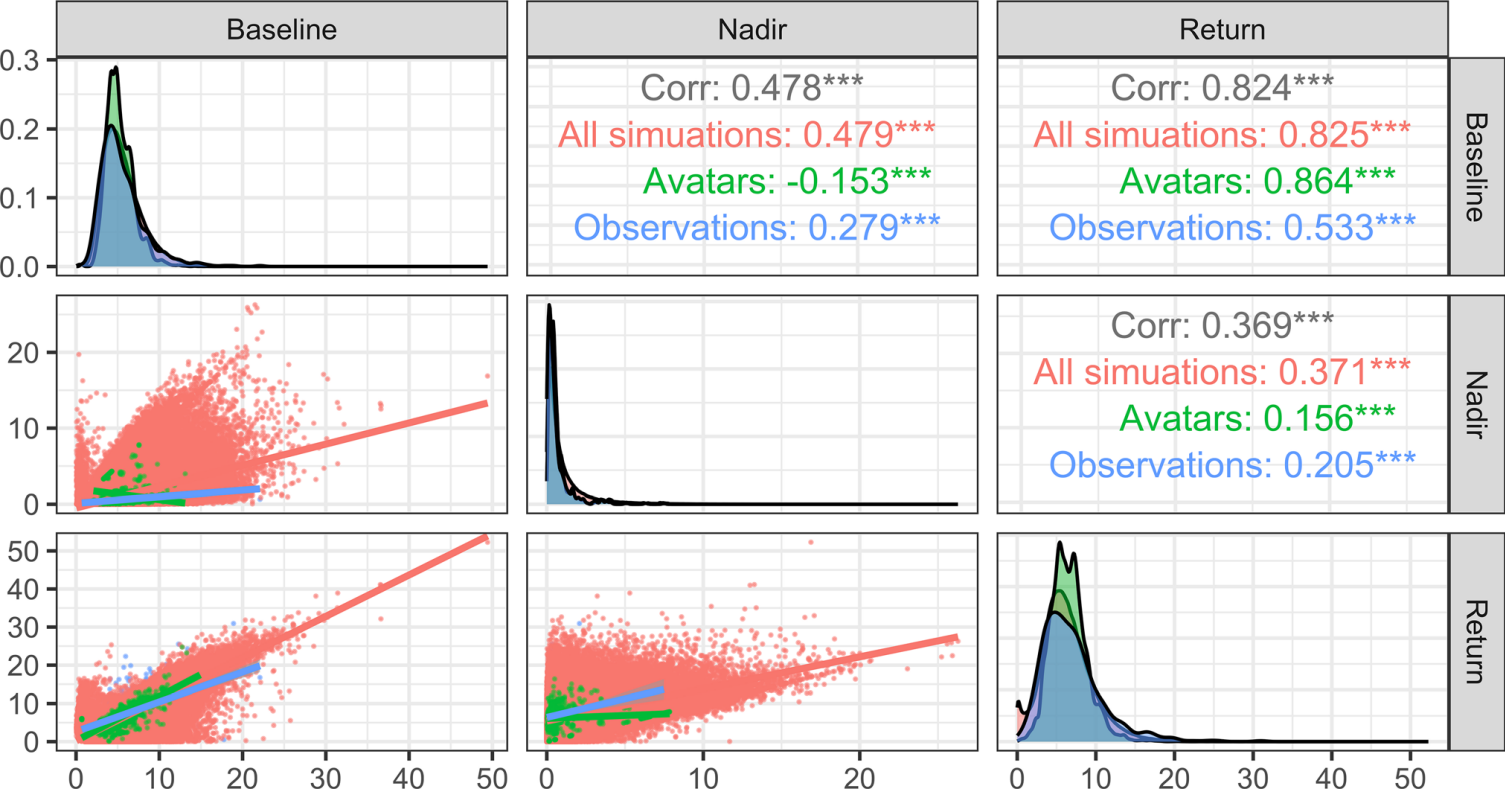
Endpoint distribution for the hematotoxicolgy model for 1000 simulations and an accuracy of agreement of ±5%. Asterisks indicates the p-value for the pearson correlation coefficient: *** for a p-value < 0.001, ** for a p-value < 0.01, * for a p-value < 0.05

**Fig. 5:**
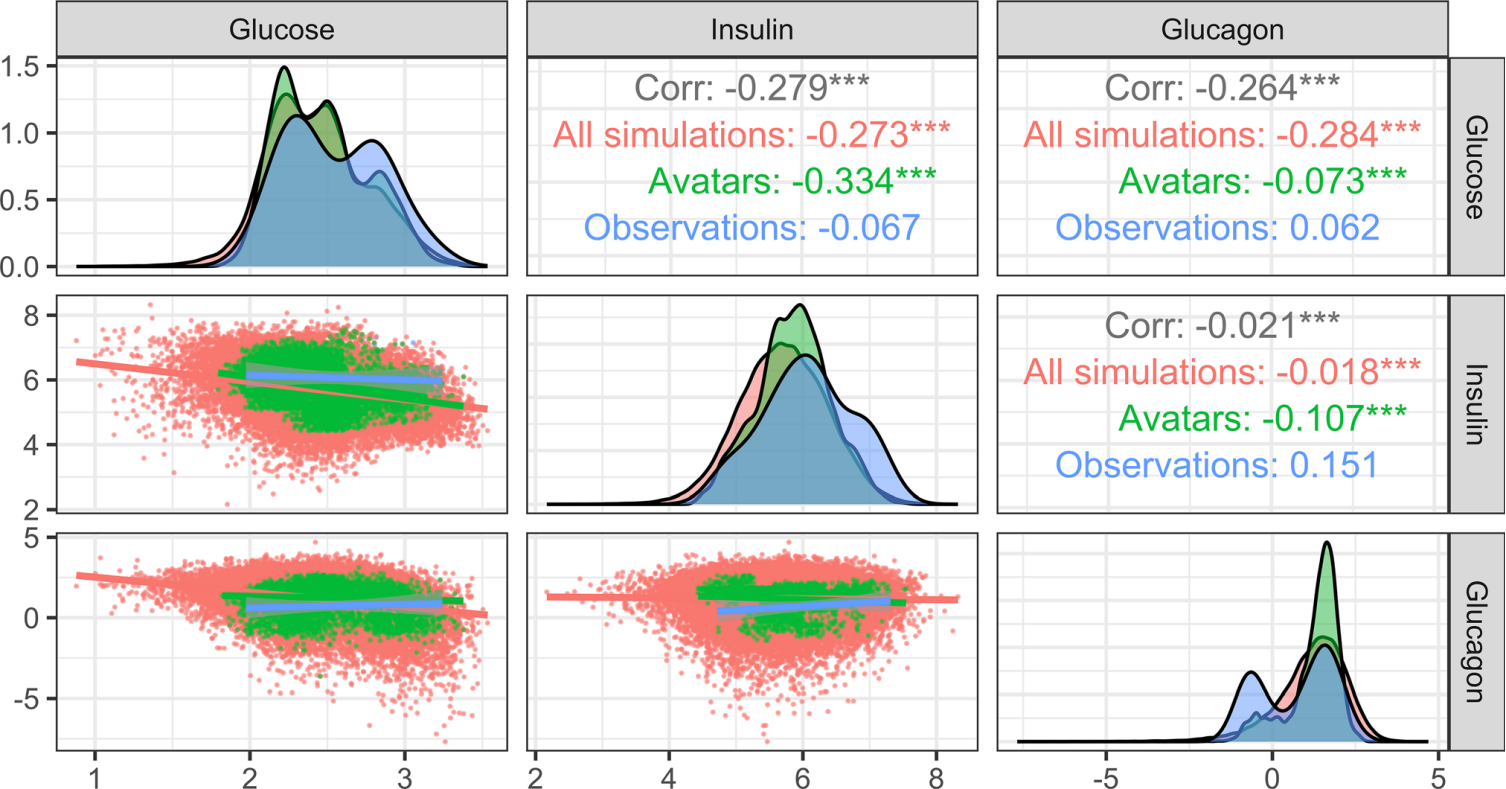
Endpoint distribution for the integrated glucose insulin model for 1000 simulations and an accuracy of agreement of ±0.29. Asterisks indicates the p-value for the pearson correlation coefficient: *** for a p-value < 0.001, ** for a p-value < 0.01, * for a p-value < 0.05

Figures 4 and 5 compare the observations to the simulations and the avatars selection using the distribution of the clinical endpoint of interest for the HT model and the IGI model respectively. The diagonal element of the plots allow to compare the marginal distribution at each clinical endpoint while the off diagonal elements allow the assessment of the correlation between the endpoints. For both models the observed, simulated and avatars distributions overlapped well and the avatars distributions were slightly narrower. In Fig. 4 the avatars did not present the first peak observed in the simulated distribution of the return endpoint, while in Fig. 5 for the glucagon endpoint the avatar distribution presented the first peak of the observed distribution while the simulated distribution did not.

**Fig. 6:**
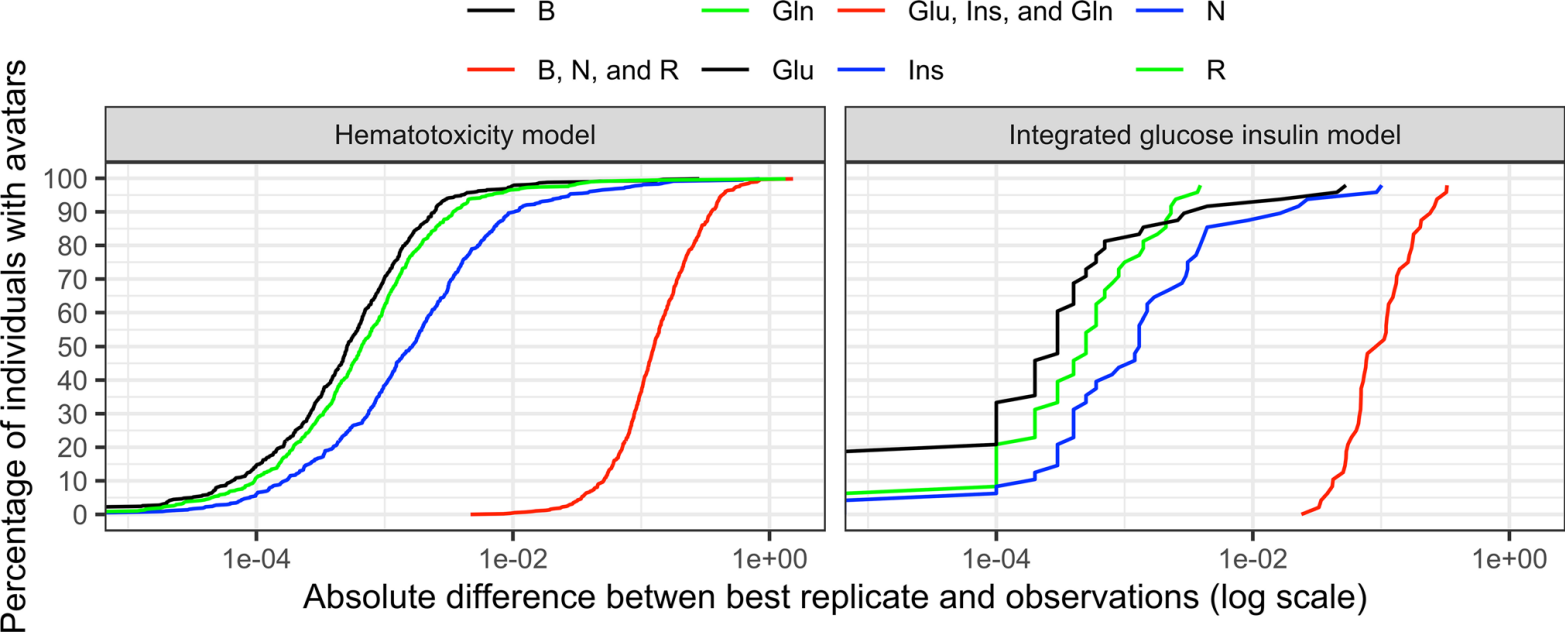
Relation between absolute individual difference between simulations and observations and percentage of individual with at least one avatar for different clinical criteria for both example models using 1000 simulations. *N* nadir, *B* baseline, *R* return, *Glu* glucose, *Ins* insulin, *Gln* glucagon

Figure 6 shows the smallest absolute difference between the observation and the individual predictions across 1000 simulations, for the clinical criterion of interest when assessing only one criterion, or for the worst criterion when assessing a combination of clinical criteria. It illustrates how close the avatars can be to the observations when using only one clinical criterion for the selection, or when using the three criteria together. Regarding the HT model (left panel), the Nadir criterion was about five times more difficult to match than both the Baseline and Return criterion individually. The absolute difference in log scale between the simulations and the clinical criteria taken separately was about $10^{-3}$, whereas it was about $10^{-1}$ when using the three clinical criteria together for a decrease of about 100-fold in accuracy. For the IGI model (right panel), the model simulations replicated more closely the glucose observations than the insulin or glucagon observations at their respective maximum observed concentrations. When considering the criteria individually, the model could replicate the observed clinical criteria with an absolute difference of about $10^{-4}$ in log scale. However, when using the three criteria simultaneously, the model could replicate the most difficult clinical criterion with an absolute difference of about $10^{-1}$ in log scale, which multiplied the inaccuracy of about 1000-fold for that clinical criteria.

**Fig. 7:**
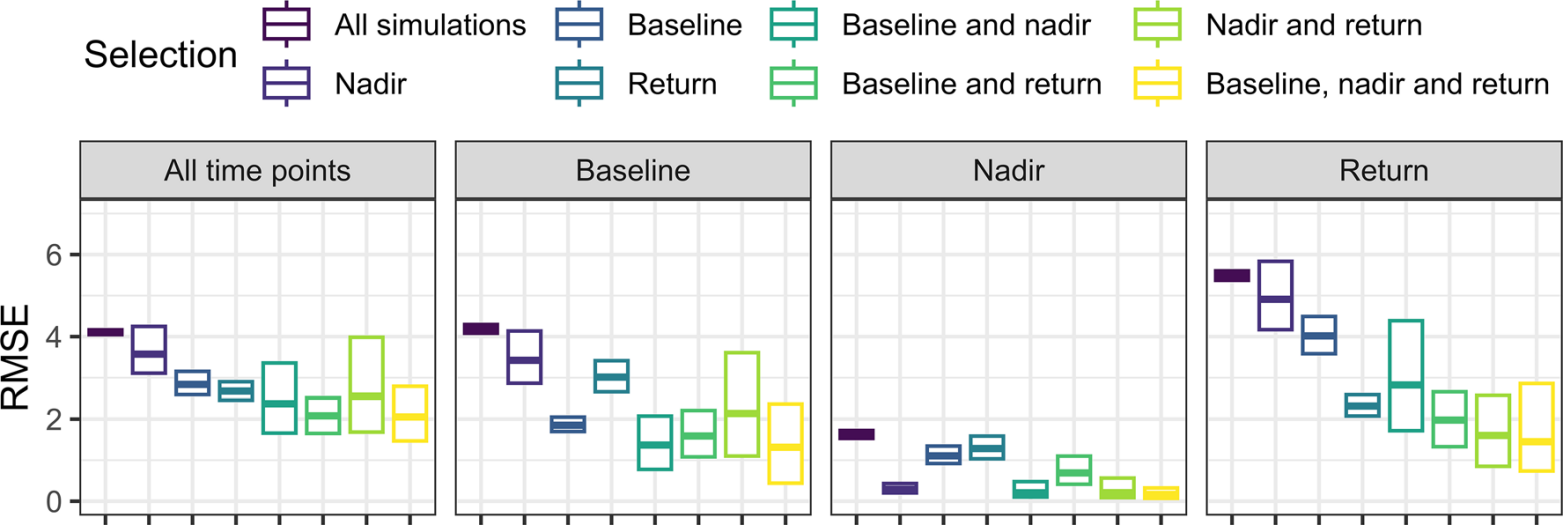
Box plot of the RMSE for avatars compared to all the simulations for the HT model (maximum number of simulations of 1000, AA of $\pm 5\%$). The plot is facetted by the time at which the RMSE was computed, and the colors indicate the clinical criteria of interest included in the selection

Figures 7 (HT model) and 8 (IGI model) show the RMSE computations using all the simulated time profiles or only the ones matching the avatar settings, at all the time points or only at the clinical endpoints of interest. For the HT model the RMSE computed over all the time points was lower for any avatars subset compared to the whole set of simulations, and the use of two clinical criteria decreased the median RMSE while the inter-quartile range was widened. Similar trends were observed for the RMSE computed at the clinical criteria of interest, with much smaller RMSE obtained when the clinical criteria of interest was included in the avatars settings. Further, when all clinical criteria were included in the avatar selection criteria the median RMSE was always lowest for the compared selection methods, but with relatively larger variance in the RMSE across replications. For the IGI model, the same trend was observed.

**Fig. 8:**
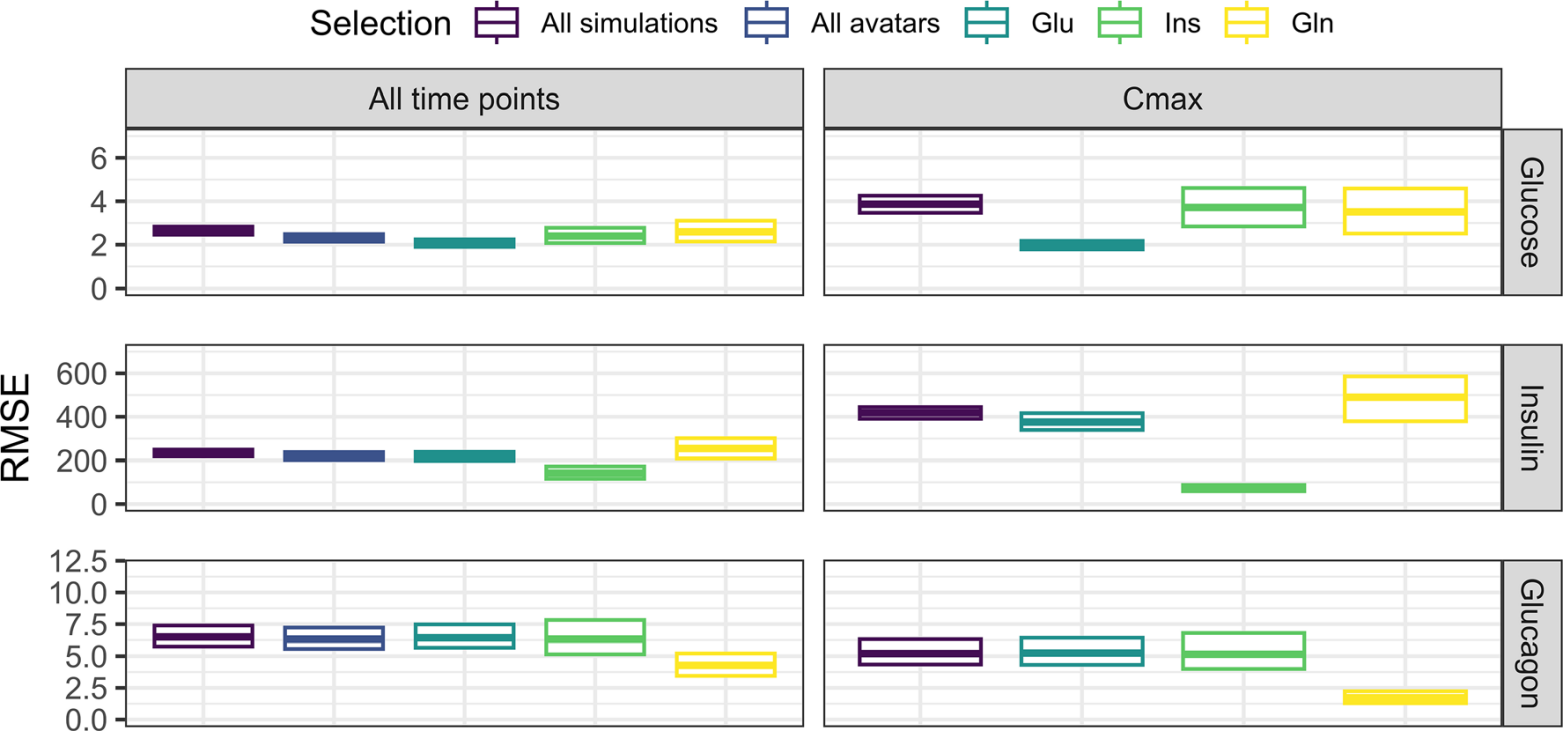
Box plot of the RMSE for avatars compared to all the simulations for the IGI model (maximum number of simulations of 1000, AA of $\pm 0.29$). The plot is facetted by the time at which the RMSE was computed and dependent variable. The colors indicate the clinical criteria of interest included in the selection. All avatars indicates that profiles not fulfilling the criteria of the dependent variable row were included in the RMSE presented if the individual was fulfilling criteria of other dependent variables (Color figure online)

## Discussion

The aim of this work was to (1) investigate methods for generating avatars with pharmacometric models, and (2) explore the properties of the generated avatars. Avatars were defined as individual simulations matching clinical criteria of interest compared to observations from a real subject within a predefined margin of accuracy. As simulations from population models have critical applications in drug discovery and development, in cases where an adequate model may not be achievable, avatars may be a better option. To investigate further these aspects, multiple avatar settings were implemented for two example models.

The results showed that, overall, for the two example models, any avatar setting succeeded in selecting simulations closer to the observed profiles than the whole set of simulations. As shown in Appendix 6, the selected time profiles were closer to the observed profiles at the time points defined as clinically relevant. This visual assessment was confirmed by the RMSE computations which were lower for any avatar settings than for the whole set of simulations for the two example models. However, for the IGI model it was not possible to find an avatar for three individuals when using the three clinical criteria with any accuracy of agreement tried, even for an avatar limited selection after 23,578 simulations. Such difficulty indicated that the model could not replicate closely this combination of clinical endpoint for these individuals. This suggests an outlier behavior for these individuals for this population model or that one might need to refine the model. The statistics about the number of avatars per subject in Table 2 and the distribution shapes from the violin plots in Fig. 2 show that an avatar setting is more difficult with increasing number of clinical criteria, stricter accuracy of agreement and lower number of simulations. The comparison of the avatar samples to the EBEs and the model theoretical parameters PDF allowed to assess whether the samples agreeing with avatars settings are a random sample from the theoretical distribution or not. The bivariates plots showed, for the HT model, that the avatars samples were in better agreement with the model parameters PDF than the EBEs for which the distribution center was shifted slightly. A similar plot was not provided for the IGI model for practical reasons as it has more than 30 parameters with IIV. The endpoint distribution was in some cases better mimicked by the avatars distribution than the simulation distribution. The plot of the best absolute difference quantified the difficulty of the chosen criteria and confirmed the increased difficulty of multiple clinical criteria in one setting. In the HT example, the nadir criteria was predicted with the largest absolute difference which can be explained by its dependence on multiple model components contrary to baseline and return. For the IGI model the $Glu_{\max}$ criteria had the smallest absolute difference which can be explained by the fact that it was the administered compound during the trial, hence more information is available to guide the predictions(i.e., dosing schedule).

According to the proposed avatars definition some settings need to be defined: clinical criteria of interest (nature and number), accuracy of agreement for each clinical criterion and generation method (simulation limited or avatar limited). Case dependent choices have to be made, impacting inevitably the results of the avatars selection. Regarding the clinical criteria of interest choices for the HT model, which describes the neutrophil count time-course after an intravenous administration of docetaxel, the baseline value, the maximal depletion and the return value of the neutrophil count were selected, which are usually used in clinical practice to monitor the myelosuppressive effect of this drug [17, 24–26]. For that example all the criteria were describing the same dependent variable. For the IGI model, the clinical criteria of interest were the maximum concentration values of three different dependent variables (glucose, insulin and glucagon). The RMSE plots showed that combining multiple criteria on a single dependent variable leads to larger decrease in RMSE than combining multiple criteria over different dependent variables. For the HT model, nadir was the criteria with the smaller impact on the overall RMSE. On a larger perspective, depending on the therapeutic area, any kind of variable predictable by the model could be used as clinical criteria for the selection, even if the two model examples focused on continuous variables. For example predicted disease severity (ordered categorical data), survival status(binary data) could also be used or $C_{trough}$. For continuous variables, in addition to the individual time point values used in the examples (e.g., Baseline, Nadir, Return or $C_{\max}$), secondary pharmacokinetic parameters could be considered (e.g., area under the curve, half-life). For models involving multiple dependent variables such as the IGI model, an avatar selection across multiple variables would assure multi-variable simulations with some agreement across the different variables.

Regarding the required accuracy of agreement for each clinical criteria to match, the choice should account for the type of dependent variable studied, and the application. Preliminary exploratory plots such as Fig. 7 with a low number of simulations (e.g., $N_s=1000$) can help to decide on the accuracy of agreement to apply for the selection of avatars by quantifying the relative difficulty of the clinical criteria of interest. A low accuracy of agreement can be decided for computational reasons, or because one really need at least one avatar per individual, whereas a strict accuracy would generate avatars closer to the observed data, but would be more expensive computationally. While our illustrations used the same required accuracy for all criteria of a model, it would be natural to vary the accuracy required according to the clinical demand. The rational can be computational constraints, knowledge that the model has good individual predictive performances for the selected clinical criteria, a low accuracy demand on criteria agreement, or the non-necessity to have avatars for every individual depending on the intended use of the avatars.

Regarding the generation method, avatars or simulation limited selection methods were explored. In both cases common problems may arise after the simulation step: either the avatar settings were too strict resulting in many individuals without avatars, or unnecessarily low accuracy leading to unachievable criteria. Avatar limited simulation selection is more interesting from a computational point of view as it allowed intensive simulations only for the individuals for which it is difficult to find avatars. This selection method also provided an the avatar simulation efficiency ratio *r* which is an individual metric of the ability of the model to replicate the clinical criteria of interest. The avatar limited selection process would also be preferred for applications were it is critical to have at least one avatar per subjects such as therapeutic drug monitoring for individualized dose adjustment. A third selection method would be to use an additional preliminary step to explore the proximity aspects of the avatars with a simulation limited selection process using a small number of simulations to identify adapted avatars settings for the main avatars selection step.

The duration of the avatar’s generation process is case dependent, mostly driven by the time to perform one simulation of the initial data set, and will increase proportionally with the total number of simulations. For the avatars limited simulations it is highly dependent on the stringency of the criteria as it might increase the number of simulations necessary to meet the criteria. Without parallelization of the process, the avatar limited setting for the three clinical criteria of interest for the IGI model was terminated manually after 6.8 days with a run time for one simulation of 25 s. For the HT model the run time to get 10,000 simulations was about 2.5 h.

Avatars differ from random simulation from the population distribution as they match some individual criteria hence they may find many applications in pharmacometrics. Diagnostics in improving the model is one, as contrary to all the simulation based diagnostic tools generated regardless of the individual observed profiles, avatars account for such information. As the avatar selection process is based on the closeness to individual profiles, it filters out simulations that are too dissimilar. At the individual level, the total number of avatar for a given number of simulations provide information about the individual agreement with the model. The avatar selection process reveals uncommon individuals for which it is difficult, or not possible, to find an avatar. These individuals may not have good individual predictive performances regarding the clinical criteria of importance. The difficulty to find an avatar may be considered as a measure of the subject uniqueness, given the model and the data. This uniqueness could be a warning for model-based dose adjustment, suggesting a more intense monitoring in term of frequency or duration. A model with good avatar performances may be more reliable for clinical trial simulation or therapeutic drug monitoring than one with poor avatar performances (e.g., therapeutic drug monitoring for antibiotics $C_{\max}$ dependent where $C_{\max}$ would be the clinical criteria used in the selection process).

Avatars are better options than random individual sampling when the model cannot give satisfactory performance with respect to a key observed endpoint, is misspecified or subject to shrinkage. Hence avatars could overcome model misspecification with respect to clinical endpoints. It could also be interesting to do clinical trial simulations with avatars only, insuring that the simulations would agree both with the model and the observed clinical endpoints. Using avatars would be a way to modify the model to improve its individual predictive performances towards some clinical endpoints of interest without model refinement.

Avatars combine the “pharmacometrician" and “clinician" view of virtual subjects by filtering samples from the model parameters PDF based on their closeness to individual observations at clinical endpoints of interest. Performing clinical trial simulations with a model having good avatar performance would be an insurance in decision making, when it comes to design of the next clinical experiment. In addition, the clinical criteria and their associated accuracy of agreement are easier to communicate to a public naïve to pharmacometrics than common assessment tools.

## Conclusion

This works explores aspects of generating avatars with pharmacometric population models and their properties. Avatars were defined as random individual parameter samples from the model distribution selected on their ability to replicate individual observations at clinically relevant endpoints within a margin of agreement. Different avatar settings were investigated (number of clinical criteria to match, and accuracy of agreement), using a hematological toxicity model and an integrated glucose insulin homeostasis model as examples. The exploration of the avatars properties showed that their time profiles were closer to the individual observation than the whole set of simulations, their sample distribution is very similar to the model distribution, and the RMSE at the clinical endpoints were improved for the avatars. Potential applications are also discussed: model refinement, clinical trial simulations, therapeutic drug monitoring, and measure of individual uniqueness.

## Figures and Tables

**Fig. 9:**
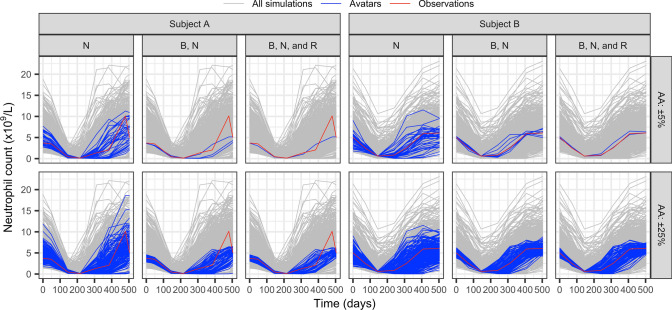
Overlay of the simulations, the selected avatars and the observations for the HT model, using a simulation limited selection with 1000 simulations for two randomly selected individuals. *B* baseline, *N* nadir, *R* return, *AA* accuracy of agreeement

**Fig. 10:**
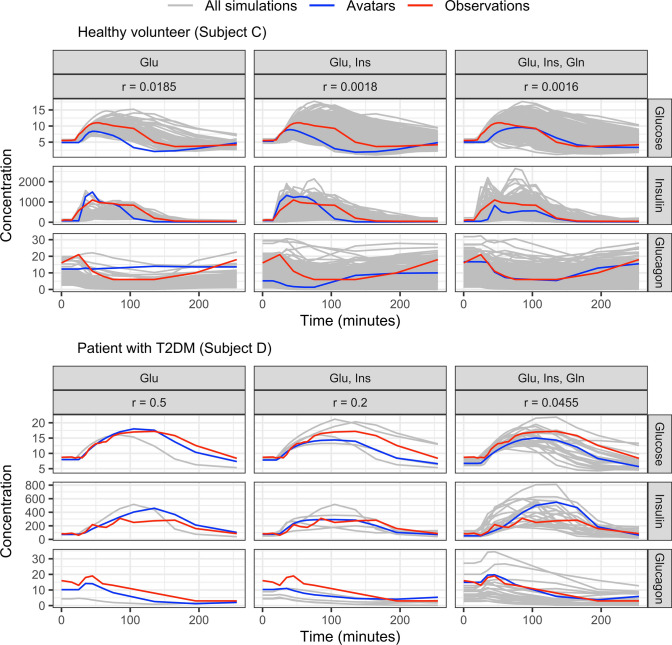
Overlay of the simulations, the selected avatars and the observations for the IGI model, using an avatar limited selection with 1 avatar for two randomly selected individuals. *Glu* glucose, *Ins* Insulin, *Gln* glucagon, *r* avatar simulation efficiency ratio

## Appendix A: Individual plots

See Figs. 9 and 10.
